# The Mellow Babies parenting programme: role of group processes and interpersonal change mechanisms

**DOI:** 10.3389/frcha.2024.1395363

**Published:** 2024-10-17

**Authors:** Jessica Tanner, Philip Wilson, Daniel Wight, Lucy Thompson

**Affiliations:** ^1^ Centre for Rural Health, Centre for Health Science, University of Aberdeen, Inverness, United Kingdom; ^2^ Institute for Public Health Science, Centre for General Practice, University of Copenhagen, København, Denmark; ^3^ MRC/CSO Social and Public Health Sciences Unit, University of Glasgow, Glasgow, United Kingdom

**Keywords:** parenting programmes, group-based interventions, group processes, mechanisms of change, maternal wellbeing, mother-infant relationship

## Abstract

**Introduction:**

Group-based parenting programmes have specific mechanisms of change compared to individual delivery. The Mechanisms of Action in Group-based Interventions framework (MAGI); distinguishes between interpersonal and intrapersonal mechanisms of change. This paper articulates a theory of change for Mellow Babies, a 14-week attachment-based group parenting programme for mothers of infants aged under 18 months, identifying the inter and intrapersonal change processes.

**Methods:**

Thirty-two semi-structured interviews were conducted with mothers and practitioners who participated in Mellow Babies, including twenty post-group interviews and nine telephone fidelity checks. Data were analysed using Deductive Qualitative Analysis based on the components identified within the MAGI framework.

**Results:**

Key interpersonal change mechanisms included: 1. Normalisation through social comparisons; 2. Validation and cognitive reframing through group feedback; 3. Peer support, offering accountability for the implementation of new habits, and providing opportunities to give and receive advice; and 4. Social and experiential learning, including internalisation of group responses leading to increased self-compassion. Intrapersonal change mechanisms were: 1. Developing new self-insight, including parenting self-awareness; 2. Increasing parenting knowledge and understanding of infant development; 3. Having time and space for self; 4. Motivation to implement new habits. Interpersonal change mechanisms had a moderating role on intrapersonal change mechanisms and subsequent programme outcomes.

**Discussion:**

The contribution of group processes and interpersonal mechanisms of change are often overlooked within programme evaluations. Findings from this study implicate their mediating role on intrapersonal change mechanisms and subsequent programme outcomes. It is important for programme deliverers and evaluators to understand the interrelationships between group processes, change mechanisms and programme outcomes to optimise efficacy and ensure cross-contextual replicability.

## 1 Introduction

Group-based parenting programmes are effective in delivering parenting support, improving parental psychosocial health and in supporting infant emotional and behavioural adjustment (1, 2). Alongside greater cost and resource efficiency (3, 4), group-based delivery may yield additional benefits compared to individual implementation: an extensive meta-analysis of 260 family support interventions found that parenting programmes offering peer-support show larger effect sizes on improving parenting skills, whereas individual home-visiting interventions generate smaller effects on child outcomes (5). Group delivery is valued by parents due to the opportunities to share experiences, seek advice and validation, normalise challenges and to expand their social support network (6–10).

Several theories and theoretical constructs can be applied to explain how group processes within parenting interventions may mediate programme outcomes:

1. Social Learning Theory (11) posits that learning occurs through observation and imitation of social models. Parents may acquire new parenting strategies from watching other parent-infant interactions and emulating them. Closer identification with the model increases the likelihood of imitation (12), suggesting social learning from peers within the group may be more pronounced than from video clips of unfamiliar parents.
2. Social Facilitation Theory *(*13) states that the presence of others can motivate greater behaviour change, due to inherent desires for positive appraisal and group approval. The group may increase parents’ accountability, and observing the progression of other parents may enhance their own effort (14).
3. Social Identification Theory (derived from Social Identity theory (15); highlights the importance of internalised social identities in shaping an individual's self-concept. Identification with others who share the same identity (for example, as struggling parents trying their best) can foster connection with others, and be protective against stigma, and increase self-esteem (16, 17). Attending a parenting group can reinforce and shape an individual's parenting identity, subsequently influencing their parenting behaviour. Social identification processes may be particularly important during matrescence (the period of transition to motherhood) when mothers experience identity loss due to the all-consuming nature of caring for an infant (18, 19).
4. Social Comparison Theory, proposed by Festinger (20) argues that social comparisons are endemic within group situations, and used by individuals to self-evaluate their own behaviour, particularly when there is close identification with other members. Social comparisons can prompt normative change, whereby individuals will shift their perspectives and behaviour based on their perceptions of “norms” for their peers. Parenting groups can normalise difficulties (6) and can also reduce maladaptive parenting strategies (e.g., corporal punishment) from exposure to conflicting thoughts and behaviours of other parents. New mothers may feel dissonance between their expectations of motherhood and their own experiences (21) and social comparisons with peers experiencing similar struggles may offer comfort and reassurance in their abilities. This in turn may override comparisons with social media portrayals of motherhood (22).
5. Social Support: Social support, including informative, instrumental and emotional support (14), is a protective factor against stress and depression (23). It may be particularly important during the transition to motherhood, which can be a time of increased vulnerability and social isolation (19, 24). Alongside mental health benefits, social support is associated with increased parental competence and better mother-infant relationships (7, 25). In addition to receiving support, parents may benefit from providing support to others, which can increase their sense of competence (26). Attending a parenting programme gives parents the opportunity to build their social support network, which may enhance their implementation of parenting strategies learnt within the group as they can seek advice and gain encouragement from their peers (5).
6. Constructivist Learning Theory: Constructivist approaches to learning (27) argue that knowledge is constructed through social interaction. Group discussions and sharing viewpoints within parenting groups support parents to ascribe new meaning to their experiences and deepen their understanding, a process guided by effective group facilitation, including active listening and motivational interviewing techniques (28, 29). Forslund et al. (30) identified two distinct learning approaches from parents attending parenting groups: those where knowledge was attained directly from programme content, and those where knowledge was co-constructed through group discussions.
7. Cognitive Reframing: Cognitive-behavioural approaches underpin many parenting programmes, supporting parents to reframe their cognitions around themselves, their child and their parenting ability. This, in turn, modifies their behaviours (31, 32). Group discussions may expose parents to alternative viewpoints, and receiving feedback from others may challenge the perceptions that they hold, increasing their self-awareness and prompting the “unlearning” of previous thought patterns and habits (33).

Understanding the contribution of group processes and the group-based mechanisms of change has largely been overlooked in evaluations of psychosocial interventions (34, 35), and there is uncertainty about the extent to which outcomes are attributable to intervention content, or to the process of group delivery: “*Is the change in parent and child behaviour attributable to the content of what is taught in the group, or is it also in the process through which the group occurs?*” [(10) pp87].

**Figure 1:**
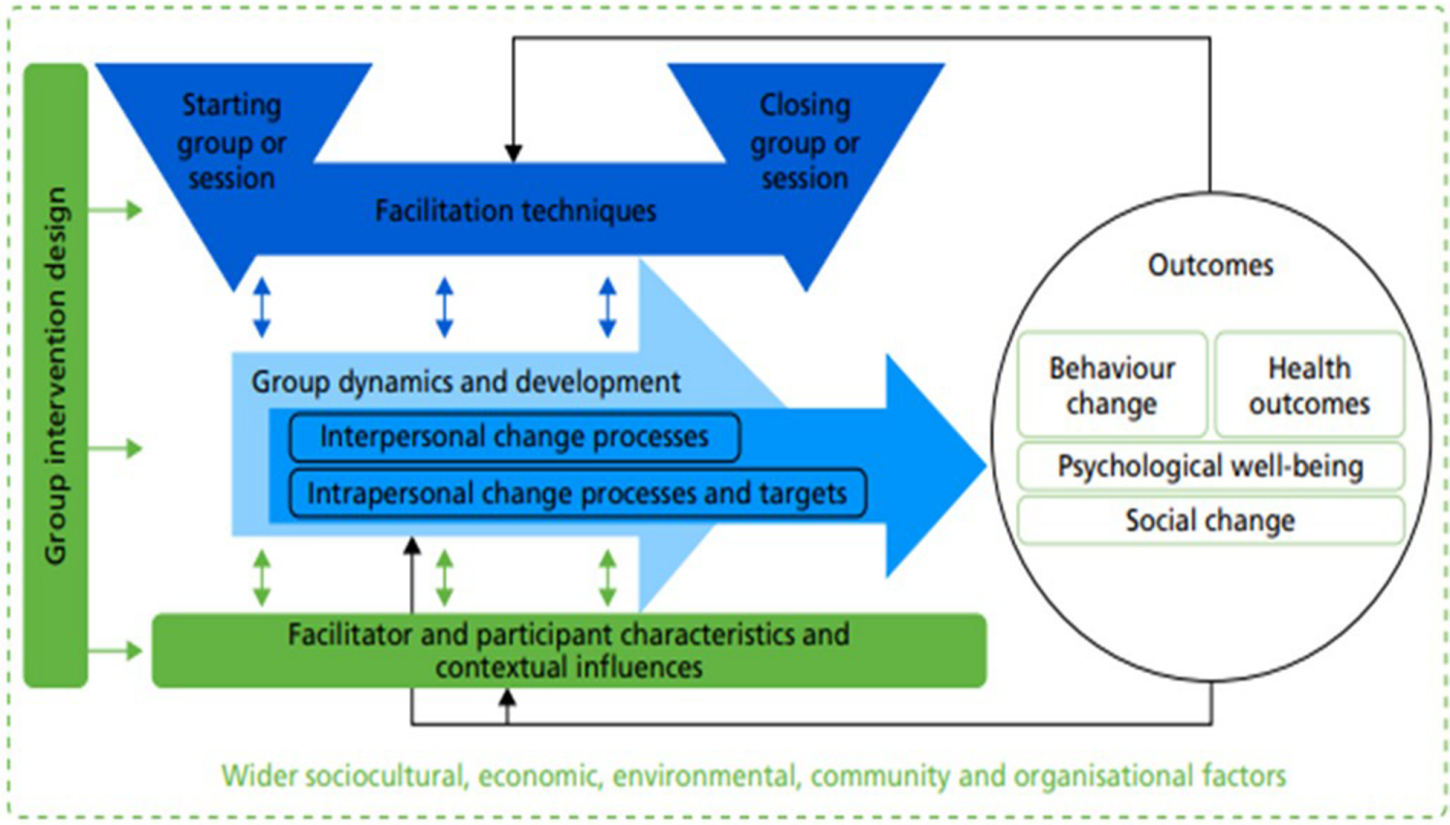
MAGI framework (taken directly from Borek et al., 2019 (34). Licensed under CC-BY 4.0.

In response to this, Borek et al. (34), developed The Mechanisms of Action in Group-based Interventions (MAGI) framework (depicted in Figure 1) which specifies the key pathways of change within group interventions. Although it was originally developed from health behaviour change interventions, the key principles can be applied to group-based parenting programmes. The framework posits that intervention and implementation approaches shape the group processes, which determine the intrapersonal (occurring at an individual level) and interpersonal (derived from group interactions) change mechanisms, in turn mediating programme outcomes. Contextual factors, including participant and facilitator characteristics, have additional impacts on programme implementation, group processes and change mechanisms.

A previous evaluation of Mellow Babies has implicated the contributions of the group elements of the programme in supporting self-reflection, reducing emotional isolation, normalising difficulties and building peer support (36). However, the relationships between group processes, interpersonal and intrapersonal change mechanisms, and programme outcomes are hitherto unexplored. In addition to determining the efficacy of a parenting intervention, evaluations should elucidate the active ingredients and change processes within an intervention to provide insight into how and why a programme works (or not) (37).

Understanding which group processes facilitate interpersonal change (38), and under what conditions (39), is necessary to optimise the effectiveness of Mellow Babies and ensure that the active ingredients are retained when delivered within different contexts. Findings would also help refine the programme's Theory of Change, a key aim of programme evaluations (37), with specific articulation of the interpersonal change processes within Mellow Babies. Due to the facilitative, group-based nature of the programme, we hypothesised that the interpersonal change mechanisms would play a significant role in determining both the intrapersonal change mechanisms and subsequent programme outcomes.

The aim of this process evaluation was therefore to explore mother and practitioner experiences of Mellow Babies with a view to understanding: 1. The interpersonal mechanisms of change, and which group processes are necessary to activate these change processes; 2. The intrapersonal mechanisms of change, and the interrelationships with interpersonal change mechanisms; 3. How the above factors influence programme outcomes. A related paper (submitted concurrently) examines how group contextual factors impact enabling group dynamics within Mellow Babies.

### 1.1 Intervention

**Figure 2:**
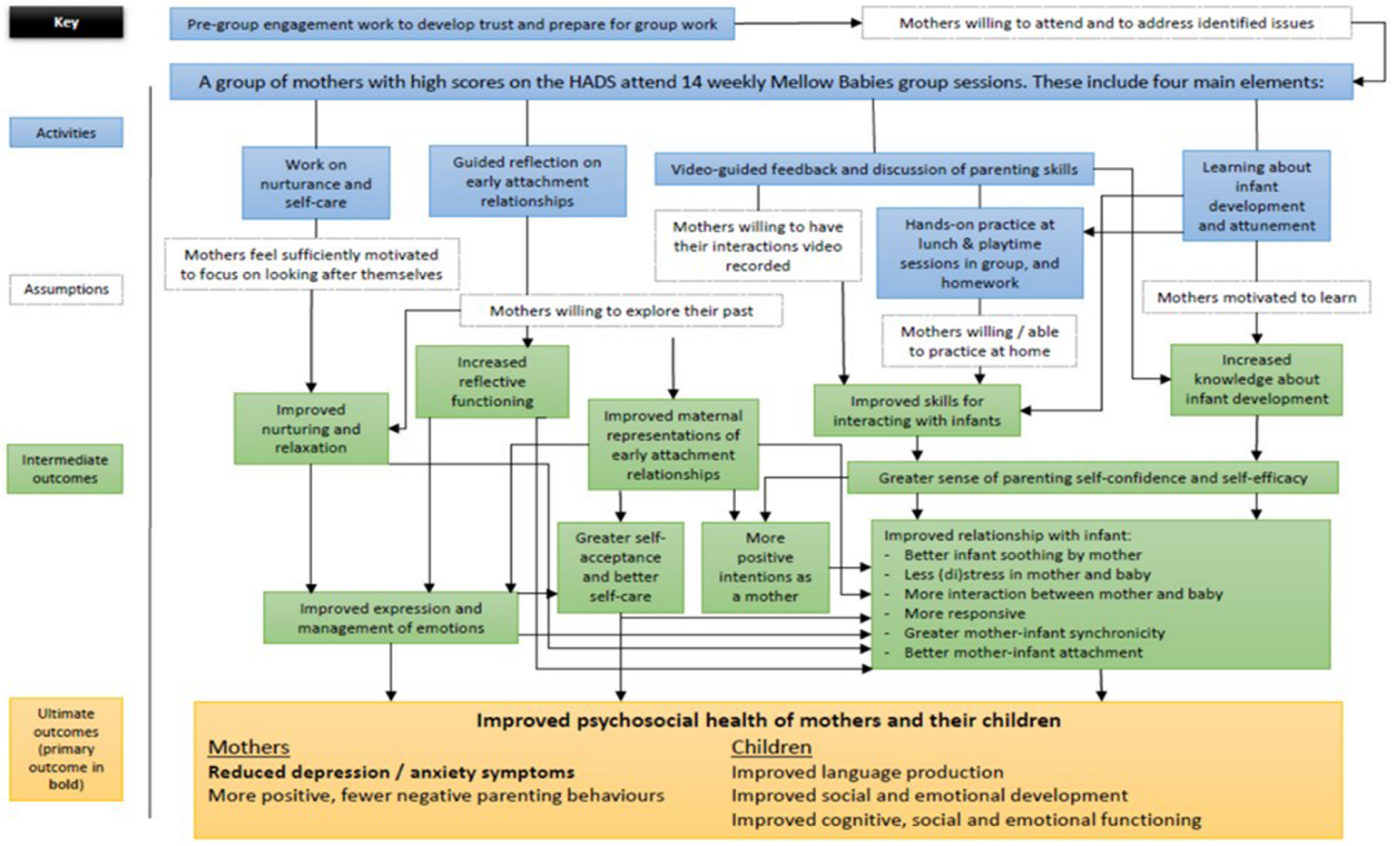
Original theory of change for Mellow Babies.

Mellow Babies is a 14-week manualised attachment-based programme for mothers of infants aged between 6 and 18 months. Groups are targeted towards mothers with existing mental health or parenting challenges, and the programme aims to improve maternal wellbeing and promote healthy mother-infant attachment. Sessions are delivered by two or three trained facilitators, who receive regular supervision during the course of the group. Each session lasts approximately five hours, with a shared lunch break that includes mother-baby activities which can be replicated at home. For the rest of the time, babies are looked after by childcare workers so mothers are able to participate fully. Sessions consists of group activities and reflective discussions, and strengths-based video feedback is used to develop maternal sensitivity and attunement. The ethos of the programme is particularly important, with groups aiming to be safe, non-judgemental, nurturing spaces which facilitate reflective discussion. Practitioners adopt a facilitative, rather than didactic approach, and a collaborative ethos is created, with facilitators participating in group reflective activities and sharing their own experiences. Preliminary evidence from pre/post evaluations and a small scale wait list trial has demonstrated positive results (40–42). The original theory of change for the programme is shown in Figure 2.

This process evaluation accompanied a Randomised Controlled Trial of Mellow Babies, conducted in the north of Scotland. However, the trial was unable to recruit to target due to social distancing restrictions enforced as a result of the COVID-19 pandemic. While quantitative outcome data were collected for all participants, the study was insufficiently powered to draw conclusions on the clinical or cost-effectiveness of Mellow Babies (43).

Mothers were referred into the trial by professionals, including health visitors and GPs or self-referred by responding to NHS Patient Identification Centre (PIC) letters and social media advertisements. Mothers were eligible if they scored above a threshold for anxiety or depression on the Hospital Anxiety and Depression Scale (HADS), were the primary caregiver of an infant aged between 6 and 18 months at the time of randomisation, and lived within the Highland Council region. Exclusion criteria included: mothers with twins or multiple births; current substance dependence; having a child with significant developmental difficulties; insufficient English to be able to participate in a group; and previous participation in a Mellow Babies group. All members of the research team were independent of programme development and delivery.

## 2 Method

### 2.1 Participants

The data presented in this paper derive from interviews conducted with seventeen mothers and three practitioners who participated in/delivered four Mellow Babies groups. All mothers and practitioners who participated in a Mellow Babies group which completed (i.e., was not curtailed by social distancing restrictions implemented during COVID-19) were invited to participate in a post-group interview. Fourteen mothers (out of a possible eighteen; 78%) opted to take part in post-group interviews (Table 1). Mothers were randomly selected to take part in telephone fidelity checks and pre-group interviews. Further detail about the Mellow Babies Trial and the demographic characteristics of participants are provided in (43).

**Table 1:** Breakdown of trial participants.

Total number of mothers recruited within trial	106
Total number of mothers randomised to Mellow Babies condition	53
Total number of mothers attending at least one intervention session	32
Total number of mothers eligible for post-group interviews (i.e., participated in groups where delivery was not impacted by COVID-19)	18
Total number of mothers participating in post-group interviews	14

### 2.2 Data collection

In order to elucidate the programme's theory of change, it was necessary to understand participants’ lived experiences of participating in and delivering Mellow Babies using qualitative approaches (44). Video recording sessions was not possible due to the confidential nature of group discussions. Thirty-two interviews were conducted in total (Table 2), including pre-group interviews (*n* = 1), telephone fidelity checks (*n* = 9) and post-group interviews (*n* = 20).

**Table 2:** Type of interviews conducted.

Type of interview	Number
Pre-group interviews with mothers	1
Telephone Fidelity checks	9
Mid-group interviews with facilitators	2
End group interviews with mothers	14
End group interviews with facilitators	6
Total	32

With the exception of the fidelity checks, which were all conducted via telephone, interviews were conducted face-to-face, via telephone, or via video-conferencing, determined by the participants’ preferences. Interviews were semi-structured, following a pre-defined interview schedule, but allowing sufficient flexibility to respond and explore relevant lived experiences of the group on an individual level. Questions aimed to elicit participants’ perceptions and experiences of the group, including how similar they felt to other mothers in the group, and which group elements of the programme they found beneficial. Interviews were recorded and transcribed verbatim prior to analysis. Ethical approval for this study was obtained from the East Midlands – Nottingham 1 Research Ethics Committee (Ref: 18/EM/0304).

### 2.3 Data analysis

Deductive Qualitative Analysis (45) was employed to analyse data. This approach begins with a conceptual framework, identified *a priori*, and used to guide analysis (46). Our analysis matrix was based on the theoretical concepts outlined in the MAGI framework (34). These concepts included contextual factors, group dynamics and development, intrapersonal mechanisms of change, interpersonal mechanisms of change, and programme outcomes. All initial concepts were preliminary, and subject to change during the analytic process (45). In this case, the contextual factors were elucidated further based on inductive findings from interviews.

Once the conceptual framework was finalised, inductive thematic analysis (47) was used to identify further sub-themes. This process was based on discussion between two authors, with the final coding framework agreed by the whole authorship team. Interview transcripts were then reviewed again, and the coding framework was refined until the themes and subthemes were considered to represent interviewees’ experiences accurately.

## 3 Results

**Figure 3:**
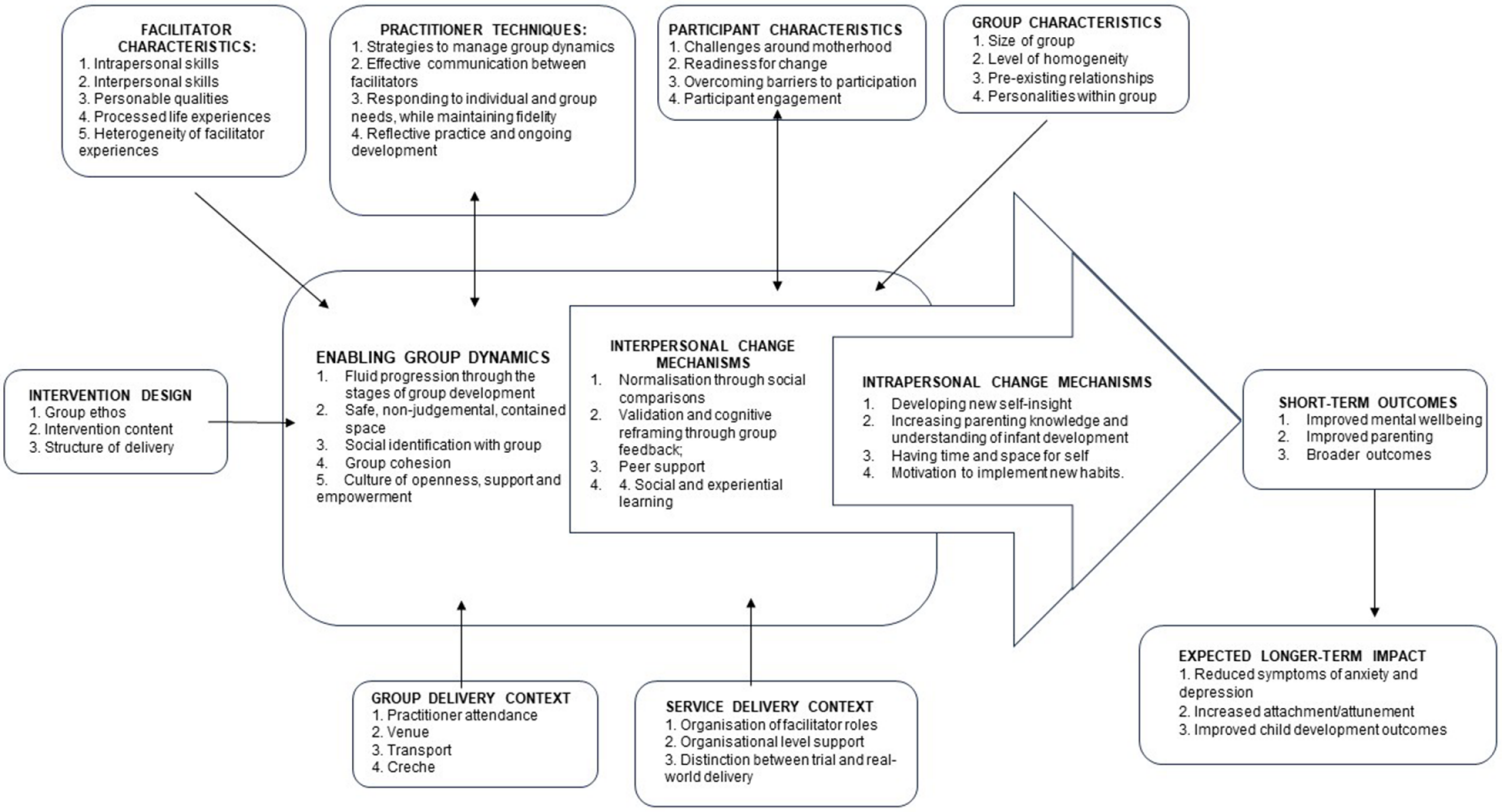
Revised theory of change for Mellow Babies, elucidating the interpersonal and intrapersonal change mechanisms.

A revised Theory of Change (Figure 3) was created, based on the concepts identified in the MAGI framework (34). This paper focuses on the mechanisms of change within Mellow Babies groups. A related paper (48) explores how group context impacts on these change processes.

As articulated in the Theory of Change, the wider context, including the group and service delivery context, and participant, facilitator and group characteristics all shape the group dynamics and development. Facilitator techniques also have a central and bi-directional impact on the group, supporting enabling group dynamics including: 1. Fluid progression through the stages of group development; 2. A safe, non-judgemental, contained space; 3. Social identification with group; 4. Group cohesion; and 5. A culture of openness, support and empowerment.

### 3.1 Interpersonal change mechanisms

#### 3.1.1 Normalisation through social comparisons

Hearing other people's experiences helped mothers appreciate the universality of life's challenges, and recognise that other people weren't necessarily having an easier time. This shifted their perspectives and gave them a re-evaluation of their own life:

*“I think I realised that my life wasn*’*t really as hard as I thought it was.”* (M17; Mother Interview)

*“I was the only single mum and they were all talking about their husbands but then it was actually coming to light more and more each week that actually their partners weren*’*t helping…Realistically they were in the same boat as me… it was just nice to know that it wasn*’*t just me that was physically alone.”* (M13; Mother Interview).

Mothers also benefitted from normalising their difficult feelings and realising that they were “*not alone”* in their struggles. It reassured them that they were “*not crazy”* or “*a bad parent”*, and alleviated the guilt and shame they felt for not always enjoying motherhood:

*“After I’ve been to Mellow Babies, after talking to other mums, you realise that all the thoughts that you have and all the feelings you have are quite normal. It makes you a bit more calm”* (M12; Mother Interview)

#### 3.1.2 Validation and cognitive reframing through group feedback

In addition to finding solace in the experiences of others, mothers also benefitted from being able to share their own stories. The group was a therapeutic space where they were able to offload and have their feelings “*heard”*, yet it was sufficiently removed from their everyday life that their concerns and frustrations could be contained within the boundaries of the session:

*“It cleared a lot of the junk out like I’ve been heard in a lot of things and that helps a lot, just to be heard. And like validated.”* (M3; Mother Interview)

There were some topics, for example, their relationship with their partner, that were difficult to talk about to people who were more involved with their life:

*“I think that was what was appreciated and also really beautiful about what the groups were doing, it was a space where we could talk about hard life stuff where we might not have people to talk about that with otherwise.”* (M11; Mother Interview)

Women described feeling overwhelmed by the validation, acceptance and support they got from the group when sharing their experiences:

*“I can*’*t explain it because I’ve never been in a situation like that before, even with my close friends, you’re there in the moment with each other and you’re processing feelings, but there*’*s this huge support round you.”* (M6; Mother Interview)

Mothers were also able to gain new perspectives on themselves and their life from the feedback of others. This included appreciating how much they had overcome in her life, and how others’ perceptions of them were much kinder than how they viewed themselves.

Mothers were able to reframe their views of healthcare professionals, realising that they were not immune from life adversity. One mother described it as “*inspiring”* that one of the practitioners had experienced similar challenges to her, and it instilled confidence that she could also pursue a career in healthcare.

#### 3.1.3 Peer support

The support proffered by the group was perceived as the most valuable element of the group by several mothers. This included encouragement within the sessions, with practitioners reporting that “*there was so much care and love in that room”,* (P1; Practitioner Interview) but also having an ongoing community of support that extended beyond the group. This support was predominantly through the WhatsApp group chat, which allowed mothers to be in touch and offer support to each other during the week. This chat gave mothers a sense of supportive accountability, as if they were implementing new strategies, someone from the group would check-in with them, and they would be able to discuss and troubleshoot any challenges. The chat also became a space where mothers were able to share and acknowledge their successes. Not only did mothers benefit from receiving support from others, it also increased their self-efficacy when they were able to give advice to others:

*“A lot of them said that they had, just speaking to other people and being able to offer tips was good to build up their confidence because it made them feel like they weren*’*t a constant failure.”* (P3; Practitioner Interview)

#### 3.1.4 Social and experiential learning

Mothers gained new insight from being around other mothers and babies, and watching their interactions. This helped them cultivate a more positive perspective on their own child, for example, appreciating that all infants develop at different rates, and recognising the developmental stage of their child through social comparisons:

“*She*’*s very much been babied., from anxiety and things. So, actually seeing her with younger babies allowed me to see her as a more independent…I’ve seen her as a next level, more as a toddler than a little baby baby.”* (M5; Mother Interview)

A further benefit of group delivery is that mothers began to internalise new ways of thinking based on the responses that they had given to others. Giving reassurance and validation to mothers who were experiencing similar struggles encouraged mothers to respond to themselves with greater self-compassion, and to implement the advice that they would give to others in that situation:

“*Sometimes a mum will text to say their child*’*s being a nightmare. I’ll quite often message back being like well you know behaviours got meaning, they’re trying to communicate something with you.,,But it*’*s also given me the opportunity to look at and do that to myself too.”* (M13; Mother Interview)

*“When you go to this group and you realise that other mums are feeling the same, you go, “Why are you making yourself feel this way? You’re brilliant, you’re doing fine.” You look at yourself and you go, “I’m doing fine too actually, I’m not that bad, I should just maybe chill out about this a little bit more,”* (M11; Mother Interview).

### 3.2 Intrapersonal change mechanisms

#### 3.2.1 Developing new self-insight, including parenting awareness

Reflective activities supported mothers to gain new insights into their past experiences, and instilled a sense of acceptance, understanding and forgiveness for some of the adversity they had faced. For some mothers, this process brought an awareness of the issues that they still needed to deal with:

*“We’re all used to our own life, and when you’re having to write your life down…it was a big turning point for me … so the group actually brought up things for me that I think I needed to deal with.”* (M6; Mother Interview)

Most mothers found it helpful to reflect on their childhood experiences and understand how they have “*followed us through our lives”*, (M1; Mother Fidelity Check) especially those who had not previously had the opportunity to do so through therapy. Mothers who had a difficult birth also benefitted from having a space to process their experiences. Many had not had the opportunity to have a debrief with their obstetrician, so had been left with a lot of questions, as well as unresolved trauma. It was perceived as particularly helpful that one of the practitioners worked part-time in a maternity unit and was able to provide further information and explanations about their experiences and why certain decisions may have been made.

#### 3.2.2 Increasing parenting knowledge and understanding of infant development

Session content helped mothers increase their understanding of child development and their repertoire of parenting tools and strategies. The majority of mothers felt the group “*taught me a lot”* and practitioners perceived that all mothers had taken some new learning from the course. The Circle of Safety activity, based on attachment theory of the parent being a secure base for exploration, was described as a “*lightbulb moment”* by several mothers, helping them to understand the value of allowing their infants to explore, and the importance of being a safe base to return to when they needed reassurance. Mothers also enjoyed learning activities which they could implement at home with their baby:

*“We get activities to go back with every week, like activities that kind of get you playing with your baby, and that*’*s been a huge difference because I think I was at the point of feeling like everything was a chore before and building up a bit of resentment to everything I had to do all the time. But it just.. it was a really simple thing, but it*’*s made a really big difference to actually sit down and enjoy playing with her.”* (M1; Mother Fidelity Check).

#### 3.2.3 Having time and space for themselves

For many mothers, Mellow Babies was the first time that they had been apart from their children. Mothers valued having time away from their children, which they described as having a “*nice break”* and how they sometimes “*need a little bit of space”:*

*“I think it was the thing I looked forward to every single week, like it*’*s my day out. It was my socialising and I think it was really good for my mental health… I think sometimes you just need to be able to go somewhere where you can chat with adults and your baby gets looked after or there*’*s somewhere for them to go, there*’*s just not enough of that.”* (M11; Mother Interview).

In addition to giving them a reason to leave the house and providing an opportunity for uninterrupted adult conversation, the group gave mothers a space that was centred on them. This was a marked contrast with other baby groups which they had attended that were focused on their children and did not yield the same benefits:

*“A lot of the time with these baby groups, you’re not really associating with the parents so much, you’re taking the kids for the kids, and most of the time you’re just focusing on the kids and things like that, so you don*’*t get that recharge.”* (M12; Mother Interview)

The majority of mothers expressed how much they valued having intellectual stimulation and time away from their infant, without experiencing any guilt because they were “*still doing something right”* (M12; Mother Interview) by their children. Being able to think about their own needs reminded them of the importance of self-care, and having conversations which extended beyond motherhood, for example about hobbies, encouraged mothers to reconnect with their sense of self that had been overshadowed during motherhood:

*“It brought me back to things that I like to do instead of just doing things with him.”* (M12; Mother Interview)

*“One thing that one of the mums kept saying was, “It*’*s great because when I come here, I’m not mum, I come here as a person, who I am, not like at home where everything revolves around the baby…I’m me, I get to say what I want and how I feel.”* (P3; Practitioner Interview)

#### 3.2.4 Implementation of new strategies

Based on their learning from session content, and the advice and reflections obtained from group discussions, mothers implemented new habits and behaviours within their everyday life. This implementation was facilitated by validation, support and accountability to the group. Key behaviours included better self-care practices, experimenting with new parenting strategies and techniques, and applying cognitive reframing strategies, such as reflective functioning techniques when their baby was having a tantrum, and improved self-compassion through internalising responses that they had given and received from the group.

### 3.3 Outcomes

The interpersonal and intrapersonal change mechanisms supported the attainment of the following programme outcomes.

#### 3.3.1 Improved mental wellbeing

Normalising their challenges, receiving validation from the group, and forming connections with other mothers supported self-acceptance, increased confidence, and reduced feelings of loneliness and isolation. Having space for themselves also helped them to reconnect to their identity, facilitating assimilation of their new identity as “mother” with their previous sense of self. Most mothers described the “*transformational”* and “*life changing”* impact the of the group by comparing how they were now to what they were like before they started group. Mothers expressed feeling “*miserable”, “depressed”* and “*the lowest, I think I’ve ever been”* prior to Mellow Babies whereas phrases such as “*content”, “uplifted”* and “*I feel back to me”* were used to describe how they now felt.

Improving their social network reduced both physical and emotional isolation, and it gave them a place where they could continue to share concerns and gain validation and support:

*“I know I always have people to talk to, whereas before, I didn*’*t have anyone.”* (M17; Mother Interview)

Feeling less lonely had a positive impact on mothers’ general wellbeing, reducing feelings of depression and improving their sense of purpose and motivation:

*“Because I felt less lonely, I felt more, it might not even make sense, but I felt like I had more energy and more willpower for things.”* (M14; Mother Interview)

Mothers felt more confident and content with themselves and their lives from normalising their struggles, recognising their own resilience, and from receiving positive feedback from the group:

*“I’m starting to value that I do have a bit more to give.”* (M5; Mother Interview)

#### 3.3.2 Improved parenting

Normalising parenting challenges, cognitive reframing from group feedback and experiential learning from observing others and through session content all improved mothers’ parenting self-efficacy, awareness, knowledge and skills. One mother stated that they were “*certainly a different mother because of the group.”* (M13; Mother Interview). Several other mothers described feeling more “*confident”* in their parenting, and able to be more “*relaxed”* with their children:

*“I feel like I have a much better relationship and much more confidence with* [baby]. *That is the biggest, actually the biggest thing and just so much more content with my mothering instincts and looking after her.”* (M3; Mother Interview)

Other specific changes included feeling a better bond with their infant, enjoying motherhood, and being able to be more present in play and general interactions. Mothers had also felt they had improved their reflective functioning and talked about reframing their cognitions when their baby was crying or having a tantrum to consider what needs they were communicating.

#### 3.3.3 Broader programme outcomes

There were also several previously unarticulated outcomes of the programme. Firstly, from practitioners sharing their life adversity, mothers shifted their perceptions of healthcare workers. Mothers described feeling surprised that their lives weren't perfect either, and practitioners felt this would have a positive impact on their future relationships with professionals, particularly mothers who had difficulty opening up:

*“How the mums have seen professionals. I don*’*t think I can underestimate how important that is, particularly for this one mum who had a lot of challenges in her life and obviously had a perception of what professionals are like. I think for her that*’*s going to be life-changing going forward. Who she goes to see now, whether it*’*s a doctor, nurse, psychologist, dentist, whatever, any profession, I think she*’*s going to see them more as a person rather than a professional. I think for her, that*’*s going to change her relationship with professionals. I don*’*t think that can be underestimated.”* (P1; Practitioner Interview)

A second broader outcome was the changes that mothers made within their lives as a result of the insight, support and motivation they gained from the group. Several mothers described the impacts of better self-care practices:

*“I’m wearing make up again, I’ve lost weight and I’m healthier’* (M13; Mother Interview).

There were also other specific steps that some mothers had taken to make positive changes within their lives which they attributed to support from the group. This included leaving a relationship which they realised was abusive, seeking further help from their GP, and taking their child to get immunisations:

*“I don*’*t know that I would have had the strength to have gone through that without the support and practical advice from the ladies.”* (M5; Mother Interview).

Finally, despite the many positive impacts of the group, some mothers found that it exacerbated some of their difficult feelings. Three mothers described feeling “*triggered”* by some of the group content which was described as “*re-traumatising”*, and two mothers stated that the programme had left them feeling worse. One mother withdrew from the programme as her PTSD symptoms had intensified as a result of session discussions:

*“I’ll go in feeling quite good and then leave feeling quite down.”* (M10; Telephone Fidelity Check).

## 4 Discussion

The revised Theory of Change for Mellow Babies emphasises the centrality of group processes in attaining programme outcomes, indicating that the benefits of group-based programmes extend further than cost and resource efficiency (3, 4, 34). Several interpersonal change mechanisms deriving from effective group interactions were identified by mothers and practitioners. Firstly, hearing others’ experiences facilitated social comparisons (20). Mothers were able to recognise that they were “not alone” in their struggles and gained a sense of comfort from normalisation of their feelings and life adversity. Having a space where they were able to be authentic about the difficulties of motherhood challenged an idealised discourse of motherhood from their perceptions of other mothers in mother-baby groups or on social media (22). Secondly, sharing their thoughts and experiences supported mothers to feel validated and accepted in their experiences. Feedback from the group supported cognitive reframing (31, 32) by providing alternative viewpoints on mothers’ experiences and shared discussions enabled co-construction of new perspectives and learning about parenthood (27). Thirdly, social and experiential learning occurred from observing and interacting with others (11). In particular, exposure to encouraging and empathetic group feedback prompted mothers to internalise supportive responses that they had both given and received within the group, increasing their self-efficacy, self-acceptance and self-compassion; Finally, the group provided a community of support which extended beyond programme sessions, reducing mothers’ feelings of loneliness and isolation, providing a forum for mothers to seek reassurance and advice, and increasing their self-efficacy through being able to support others (7).

Intrapersonal change mechanisms were underpinned by both session content and the interpersonal change mechanisms. Although some degree of intrapersonal change could be attributed solely to programme content, including the “Circle of Safety” model of attachment and exposure to an increased repertoire of play-based activities, intrapersonal change processes could all be moderated by effective interpersonal change: The intrapersonal change mechanism of greater self-reflection and acceptance of their experiences could be enhanced through group discussions and feedback, alongside individual contemplation of reflective prompts within sessions; Parenting skills and knowledge were developed through didactic programme content, but enriched through social learning and advice; Having a break from childcare and having a space centred on their needs helped mothers reconnect to their previous identity and integrate their new identity as a “mother”, but this was also supported through authentic, meaningful and uninterrupted conversations with others in a similar situation (16). Finally, mothers implemented new habits and behaviours, a process which could be facilitated by the support, motivation and accountability offered by the group, akin to Social Facilitation Theory (13).

Interpersonal and intrapersonal mechanisms of change determined self-reported programme outcomes. Mothers reported improved mental wellbeing, including greater self-acceptance and self-confidence, reduced isolation, improved motivation, and feeling more connected to their sense of self. They also reported positive changes in their parenting, including improved parenting self-efficacy, reduced parenting anxiety, a closer bond with their child, and greater enjoyment of spending time together. Perceptions of healthcare professionals had shifted following the group, in line with findings by Davidson et al., (36), and practitioners felt this “humanisation” would improve mothers’ relationships with professionals in the future. Mothers implemented better self-care practices and several described making significant life-changes as a result of encouragement from the group.

Although most mothers described positive benefits from the group, a few found content “*triggering”* and two described feeling “*worse”* following the group. This corresponds with findings from other parenting programmes which suggest group delivery is not universally beneficial (49–51). Parents may be “triggered” by others parents’ stories, particularly if they have not come to terms with their own childhood experiences (50, 52). Adverse programme effects are often overlooked (53), despite 5% of group therapy participants reporting negative impacts of treatment (54). There are requests for adverse impacts to be reported and articulated within programme Theories of Change (55, 56) to aid understanding of the populations and group contexts under which programmes are most or least effective (39).

Contextual factors at a micro and macro level affect programme implementation (57), and a range of implementation factors, such as facilitator training and support, recruitment procedures, and strategies to enhance programme engagement, can all affect group dynamics and development, alongside parent, practitioner and group characteristics. These are discussed in more detail in a related paper (under review). In particular, practitioner techniques support positive group dynamics and development, which enable the above change processes. The group progressed through the stages of development outlined by Tuckman (58): forming, storming, norming, performing and adjourning. These early stages of group development could be inhibited by low levels of parent engagement, including low attendance and participation. A safe, non-judgemental and empowering culture, and the development of group cohesion through sharing vulnerabilities helped the group transition into the “performing” phase where therapeutic change can occur. Maintaining contact and support between group sessions through meeting up and group messaging facilitated group cohesion.

### 4.1 Implications

Findings support wider consideration of the group processes and interpersonal change mechanisms when evaluating group-based programmes. Greater understanding of the interrelationships between group dynamics and development, interpersonal change mechanisms, intrapersonal change mechanisms and programme outcomes is needed both within specific interventions and at a general level (34, 35). Understanding which group processes are necessary to promote interpersonal change (38) et al., and how these in turn interact with intrapersonal change mechanisms is essential for optimising programme efficacy (37) and for understanding what works, for whom, in what circumstances (39). Qualitative process evaluations, incorporating the viewpoints of multiple stakeholders, are necessary to identify potential moderating and mediating variables and to elucidate programme mechanisms of change, and these should be conducted alongside quantitative evaluations to deepen understanding (37, 59–61). Programme Theories of Change should distinguish between interpersonal and intrapersonal change mechanisms and stipulate their inter-relationship. It may also be helpful for Theories of Change to articulate adverse outcomes, whom they affect, and under what contextual conditions (55, 56).

The likely impact of group processes on programme outcomes suggests that programme deliverers may benefit from paying greater attention to the group. Peled and Perel (62) pp397 advocate the need for “*dual-attentiveness”* during group delivery, with practitioners focusing on responding to and guiding group processes, in addition to delivering session content. Practitioners may benefit from programme training which provides a comprehensive understanding of relevant group theory and how group interactions can support and induce therapeutic change (29). Regular supervision from experienced practitioners will help cultivate awareness of group processes and develop strategies to create an enabling group environment and manage challenging group dynamics. New practitioners may benefit from co-delivering with more experienced group facilitators who can share their insights into the group processes, and articulate and model “*dual-attentiveness”* techniques. Programme manuals should also include content to support practitioners to attend to, and effectively guide, group processes (62).

### 4.2 Limitations

This process evaluation was conducted alongside a randomised trial of Mellow Babies. The applicability of efficacy trials to real-world delivery has been questioned [e.g., (50, 63, 64)], and indeed there were several elements of this study which were not representative of community delivery. Firstly, due to the 14-week intensity and high programme costs, the sample of participants had significantly lower levels of need than mothers attending groups outside of the trial, who are often referred to the programme due to the involvement of child protection services. Secondly, practitioners were recruited and trained specifically for the trial, which led to difficulties with recruitment, retention and wider-organisational support, something common within trials of group-based programmes (38). Practitioners were not only inexperienced in delivering Mellow Babies, but some had little or no prior experience delivering group-based therapeutic or behaviour change interventions. Finally, Mellow Babies existed as a standalone programme, whereas in a real-world setting, it would often be embedded within wider community services, and parents and practitioners would benefit from more extensive support. All of the above limit the generalisability of findings, and further research is needed into group processes and change mechanisms within an effectiveness trial of real-world delivery. Interviews could also be conducted with a wider range of stakeholders, including programme purveyors, supervisors and trainers, to improve the validity and applicability of the Theory of Change.

As articulated in the revised Theory of Change, contextual factors at both a macro and micro level influence programme implementation, affecting the group dynamics and development, and subsequently shape programme outcomes (57). Programme fidelity deemed an essential consideration within programme evaluations (65), has also not been taken into account.

Finally, the outcomes identified within this paper are based on the self-reported outcomes identified by mothers within interviews. Future study should explore the impacts of group processes and interpersonal mechanisms of change using quantitative outcome measures. Conducting longer-term follow-ups with mothers will also help assess whether change mechanisms are associated with sustained change in wellbeing and parenting behaviours.

## Conclusions

This article articulates a revised Theory of Change for Mellow Babies, based on the MAGI framework (34), elucidating the interpersonal and intrapersonal mechanisms of change. Group elements, including the within-group processes and ongoing peer support, were deemed to be the most valuable aspects of the programme and were pivotal in determining outcomes. Key interpersonal change mechanisms within the programme are: 1. Normalisation through social comparisons; 2. Cognitive reframing through group feedback and constructivist learning 3. Peer support, offering accountability, and providing opportunities to give and receive advice; and 4. Social and experiential learning, including internalisation of group responses leading to increased self-compassion. The effectiveness of essential programme content, for example, the Life Stories session, was dependent on the interpersonal change mechanisms of normalisation, validation, and peer support.

The contribution of group processes and interpersonal mechanisms of change are often overlooked within programme evaluations (34, 35). Findings from this study implicate their mediating role on intrapersonal change mechanisms and subsequent programme outcomes. It is therefore vital for programme deliverers and evaluators to understand the interrelationships between group processes, change mechanisms and programme outcomes to optimise efficacy and ensure cross-contextual replicability.

## Data Availability

The raw data supporting the conclusions of this article will be made available by the authors, without undue reservation.
